# Editorial: Advances in identifying individuals at clinical high risk (CHR) for psychosis: perspectives from North America

**DOI:** 10.3389/fpsyt.2023.1357838

**Published:** 2024-01-16

**Authors:** Sarah I. Tarbox-Berry, Daniel J. A. Devoe, Rishab Gupta

**Affiliations:** ^1^Department of Neurology, School of Medicine, Wayne State University, Detroit, MI, United States; ^2^Department of Psychiatry, School of Medicine, Yale University, New Haven, CT, United States; ^3^Department of Psychology, Mount Royal University, Calgary, AB, Canada; ^4^Department of Psychiatry, Cumming School of Medicine, University of Calgary, Calgary, AB, Canada; ^5^Department of Psychiatry, Harvard Medical School, Boston, MA, United States; ^6^Department of Psychiatry, Brigham and Women's Faulkner Hospital and Harvard Medical School, Boston, MA, United States

**Keywords:** clinical high risk, psychosis, trauma, stress, environmental risk, social functioning, treatment, prodrome

The goal of this Research Topic is to present current work and insights from psychosis-risk research initiatives based in North America. It is well-established that the risk of psychosis onset is highest between mid-adolescence and early adulthood. Accurate, early identification of young people at high risk of developing psychosis [“clinical high risk” (CHR or CHR-P)^1^] affords the critical opportunity to provide interventions aimed at mitigation or prevention of psychosis. Although identification of risk is foremost based on clinical diagnostic criteria (e.g., unusual thought content, suspiciousness, and perceptual abnormalities) established using structured interview-based assessment tools,^2^ there is increasing attention on how cultural, demographic, and environmental contextual factors influence risk of psychosis, contribute to symptom presentation, and inform effective treatment development and delivery.

The five articles contributing to this Research Topic address three timely interconnected themes: (1) Culturally- and environmentally-informed symptom assessment, diagnosis, and treatment, (2) The relationship between exposure to contextual risk factors, stress, and clinical presentation, and translation into treatment development and delivery, and (3) Stress management, coping skills, and social functioning as targets for intervention.

Bridgwater et al., Devoe et al., and Zarubin et al. all examine the role of contextual factors in risk identification and symptom presentation. Bridgwater et al. argue that the inclusion of contextual factors in risk assessment is essential for accurate, unbiased determination of vulnerability to psychosis, and excluding these factors may lead to assessment bias and misdiagnosis. They present a narrative review of eight contextual factors relevant to CHR assessment in North American populations: race/ethnic identity, experience of discrimination, neighborhood context, trauma exposure, immigration status, gender identity, sexual orientation, and age. Taking each factor in turn, Bridgwater et al. present current research addressing contextual effects on risk of psychosis. The authors then offer practical clinical guidance for incorporating these contextual factors into assessment protocols.

CHR youth are highly vulnerable to the emotional and physiological effects of stress, including environmental stressors, interpersonal stress, and trauma. Stress exposure exacerbates CHR symptoms (1), and physiological changes in response to stress have been shown to precede psychosis onset (2). Experience of trauma is reported by the majority of CHR youth (3), including childhood, environmental, and systemic sources of trauma. Given the consequences of stress exposure, it is not surprising that a history of trauma is associated with a greater risk of transition to psychosis among CHR youth (4). In their perspective piece, Zarubin et al. focus on the characterization, assessment, and treatment of trauma in CHR youth, including key and often overlooked considerations regarding developmental timing, frequency, and intensity of trauma exposure. They note that trauma can come from multiple and often overlapping sources, including childhood trauma, exposure to crime and violence, population density, and poverty, and discuss the unique needs of CHR youth vs. adults with schizophrenia.

Devoe et al. examine how early contextual/environmental exposure and premorbid adjustment may relate to the clinical presentation of CHR youth. In their article, Devoe et al. focus on persistent negative symptoms (PNS) in CHR youth, specifically social anhedonia, avolition, and decreased expression of emotion. They present original research examining premorbid adjustment, life events, history of trauma, bullying, cannabis use, and emergency or inpatient treatment utilization in CHR youth with and without PNS. Primary results show significantly lower child and adolescent premorbid adjustment among the sample of CHR youth with PNS vs. the non-PNS CHR sample. Additionally, worse premorbid adjustment in late adolescence predicts PNS independent of other variables. These results are consistent with the substantial evidence that low social functioning and limited connection with peers, particularly during late adolescence, predicts greater symptom severity and higher risk for transition to psychosis (5).

Late adolescence through early adulthood is a period of consequential neurodevelopmental change (6) and increased exposure to instrumental and interpersonal stress. As noted by Gupta et al. and Bargiota et al., this period offers an opportune window for intervention, yet as cautioned by Zarubin et al., also presents unique challenges such as changing environmental stressors and shifting treatment targets.

Treatments aimed at mitigating the impact of exposure to environmental risk factors and improving clinical outcome in CHR youth are being developed and tested. Gupta et al. introduce the Skills Program for Awareness, Connectedness, and Empowerment (SPACE) that they are currently developing for CHR youth ages 13–18. This program targets specific sources of functional impairment and heightened distress in CHR youth that are thought to contribute to symptom progression. The 21-week skills group is organized into three successive stages building on the skills developed during the previous stage(s): (1) coping and stress management, (2) self-concept and identity formation, and (3) interpersonal connectedness and communication.

Bargiota et al. examine a neurophysiological-based approach to enhance social cognition in CHR youth utilizing intranasal oxytocin (OT). Bargiota et al. review six studies presenting results of five randomized controlled trials that investigated effects of intranasal OT in CHR or early psychosis adolescents and adults. Four studies (three trials) focused on CHR samples, with results indicating OT-induced changes in brain activation during completion of social cognition tasks and changes in autonomic activation (stress response). These results suggest that oxytocin may affect processes relevant to social cognition and stress sensitivity in CHR youth, but as Bargiota et al. point out, substantial work is needed before impact on clinical symptoms is known.

In the field of early identification and intervention in psychosis, there is increasing emphasis on the role of contextual factors in risk of psychosis and their importance for achieving the goals of improving early identification, decreasing symptom severity and distress, and minimizing risk of psychosis. Together, the articles included in this Research Topic examine the nature and impact of contextual factors, consider issues of assessment and characterization, and discuss context-informed treatment strategies.

## Author contributions

ST-B: Writing – original draft, Writing – review & editing. DD: Writing – review & editing. RG: Writing – review & editing.

## Footnotes

^1^Also referred to as “Ultra-High Risk” (UHR) in the literature.

^2^For research conducted in North America, the Structured Interview for Psychosis-Risk Syndromes (SIPS) is the gold standard for diagnosis.
